# Benefits of being part of a support group for caregivers of children with multiple disabilities: a qualitative study

**DOI:** 10.17843/rpmesp.2024.411.13218

**Published:** 2024-03-27

**Authors:** María Elena Rodríguez-Vélez, Claudia Patricia Cantillo-Medina, Alix Yaneth Perdomo-Romero

**Affiliations:** 1 Universidad Surcolombiana. Facultad de Salud, Programa de Enfermería. Huila, Colombia. Universidad Surcolombiana Universidad Surcolombiana Facultad de Salud Programa de Enfermería Huila Colombia

**Keywords:** Self-Help Group, Disabled Children, Social Support, Community Nursing, Qualitative Research

## Abstract

**Objective:**

. To explore the perceived benefits of participating in a support group of caregivers of children with multiple disabilities.

**Materials and methods.:**

A qualitative study with a focused ethnographic approach was conducted from October 2022 to February 2023, in which we applied convenience sampling. We included 20 caregivers of children with multiple disabilities. Information was collected through participant observation, focus groups, and semi-structured interviews. Thematic analysis was performed by applying Braun and Clark’s proposals.

**Results.:**

The emerging themes were: social support network: integrating, informing, and helping each other; learning space: learning to take care and to take care of oneself; promoting empowerment: identifying and facing access barriers.

**Conclusions.:**

We found that the support group functions as a social support network provides information, reduces uncertainty, and facilitates coping and resilience after the birth and upbringing of a child with multiple disabilities. It is a space where one learns to care for and take care of oneself and where empowerment for the defense of the rights of children with disabilities is promoted.

## INTRODUCTION

The probability of a child having a disability before reaching the age of five is ten times greater than the probability of dying ^1^. Children with multiple disabilities exhibit various expressions of impairment, always associated with motor and intellectual deterioration, placing them in a position of high dependency ^2^. Childhood disability poses a significant challenge globally ^3^, recognized as a public health and social issue, with repercussions on the quality of life for millions of children and their caregivers ^4^. Worldwide, a considerable number of children are estimated to face some form of disability, impacting their ability to fully engage in society and access appropriate educational, health, and social opportunities ^5^.

In Latin America, including Colombia, the situation of childhood disability presents specific challenges. While there have been advancements in terms of legislation and inclusion policies, many families struggle to access adequate health and education services for their children with disabilities. Moreover, discrimination and social stigma remain significant barriers affecting the daily lives of these children and their caregivers ^3^. In Colombia, Huila is among the departments with the highest proportion of people with disabilities, with children representing 8% of this population ^6^.

Children with disabilities are disadvantaged in most measures of child well-being, have limited access to health and rehabilitation services in low- and middle-income countries ^7^, and there is a shortage of trained healthcare professionals to cater to them ^8^. In fact, in our country, despite having a Public Policy on Disability and Social Inclusion ^9^, there are persistent difficulties in accessing basic education, health, and social protection services for these children, exacerbated in recent years due to the COVID-19 pandemic ^10^.

The family has a crucial role in ensuring the development of children with disabilities ^11^ but faces various psychological, social, and economic pressures ^12^ that increase if the child has multiple disabilities ^13^. The psychological impact is greater on women, who must deal with separations or divorces, take on the exclusive role of caregivers, and endure social stigma ^14^; they feel inferior, insecure, and stressed due to economic difficulties ^15^; they exhibit clinically significant depressive symptoms ^16^ and sleep disorders ^17^; feelings of guilt, loss of opportunities, maternal sacrifice, worry about the future, and lack of socioeconomic support are common ^18^.

Support groups (SGs) are an alternative social support network; they promote understanding of the situation and caregiving skills, reduce social stigma and isolation ^11^, provide information, and emotional and relational support for women with children with disabilities ^19^, promote resilience, family coping, health, and well-being of caregivers ^20^. Recently, they have been considered in our country as part of the empowerment component of the Community-Based Rehabilitation (CBR) Strategy for the social inclusion of people with disabilities ^21^. Despite these benefits, evidence indicates that parents of children with disabilities have restricted support networks or lack them, compared to parents of children without disabilities, thus various research studies urge healthcare professionals towards the need to support or implement such strategies ^22^.

Additionally, there is a shortage of research specifically focused on the perceived benefits of SGs for caregivers of children with multiple disabilities in Latin America, including Colombia. Although studies are exploring the impact of SGs on caregivers of children with disabilities in general, a detailed understanding of how these groups specifically benefit those caring for children with multiple disabilities in developing countries like Colombia. Given the above and the establishment of an SG for caregivers of children with multiple disabilities in southern Colombia, which has remained active for six years and has evolved to be recognized at the municipal and even departmental level, an ethnographic qualitative study was conducted to explore the perceived benefits of participating in a support group for caregivers of children with multiple disabilities, aiming to understand how these dynamics influence their caregiving experience and well-being.

**KEY MESSAGES**

Motivation for the study: Filling a knowledge gap regarding support groups for caregivers of children with multiple disabilities.

Main findings: Support groups are valuable spaces for social support and learning for caregivers of children with multiple disabilities. They facilitate resilience and coping strategies following the birth of a child with multiple disabilities. They promote the participation and empowerment of caregivers of children with multiple disabilities to address access barriers and advocate for the fundamental rights of children.

Implications: It is important to promote and support support groups for caregivers of children with multiple disabilities.

## MATERIALS AND METHODS

### Study Design

Focused ethnography study, a contemporary design focused on the small elements of society and the lives of individuals who are “socially and culturally fragmented and differentiated”; centered on studying the shared experiences of a more limited and predetermined phenomenon within small social groups to understand specific social issues, the interrelationship among individuals, and the social context in which participants live and share their perspectives on social events and issues ^23^.

The information was collected from October 2022 to February 2023 in southern Colombia, with a support group of caregivers of children with Multiple Disabilities who are part of a support network called “Disability Huila.” The first step in the research was to establish contact with the network leader, who acted as the gatekeeper ^24^.

### Research Team

The research team consisted of three investigators with experience in studies using qualitative designs. The investigators were affiliated as faculty members of Universidad Surcolombiana at the time of the study.

### Context

The “Disability Huila” support group has been a vital initiative in the region for six years. It emerged in response to the growing need for support and guidance for caregivers facing the unique challenges associated with raising children with multiple disabilities in the local context. It was founded in 2018 by a small number of caregivers seeking to share experiences, and resources and provide mutual support. The support group has experienced significant growth in both size and recognition, expanding from an informal group to a well-established and recognized support network at the municipal and even departmental levels.

Currently, the support group is comprised of approximately 30 dedicated and active caregivers who play a vital role in the community of caregivers of children with disabilities in the Huila region. It has had a significant impact on the local community by improving the quality of life of its members and raising awareness among governmental authorities about the needs and challenges faced by families of children with disabilities in the region.

### Participant selection

Convenience sampling was conducted ^25^, involving 20 caregivers of children under 9 years old with multiple disabilities, who met the criteria of being over 18 years old, having been members of the support group for a minimum of four years, and voluntarily agreeing to participate in the study. There were no individuals who refused to participate in the study.

### Data Collection

To characterize the participants a form was used. The researchers interacted with the participants’ environment to observe their activities in their natural context. Additionally, they were invited to a university classroom to form two focus groups, each consisting of 10 caregivers, with an approximate duration of 90 minutes. The purpose was to conduct an initial immersion and exploration of collective viewpoints on the topic and to define, in conjunction with a literature review, the themes for the semi-structured interviews. Subsequently, twenty semi-structured interviews and participant observation ^26^ were conducted at the support group’s meeting place by the researchers experienced in the defined methodology’s data collection techniques. No repeated interviews were conducted.

Before obtaining the information, participants were briefed on the methodology, and their consent was sought to participate in the study and record the audio of the interviews. The following was the guiding question: “What are your opinions on the functionality of the support group, your experience in the group, and the perceived benefits?” From this question, discussion, and discourse construction were generated by the group on the topic of “benefits of belonging to a support group”.

In addition to analyzing the information obtained from each focus group, participants were individually interviewed to further explore and delve deeper into specific emerging topics of interest. This served as an alternative data triangulation method, contributing to richer, broader, and more in-depth information, ultimately enhancing the credibility of the results. Interviews were conducted until data saturation was reached, indicating no new significant findings were emerging. All focus group discussions and interviews were audio-recorded and transcribed in full within 48 hours. As described, the transcribed interviews were returned to the participants for their review and approval.

Participant observation ^26^ was conducted gradually by the lead researcher. Initially, the researcher observed from a certain distance, but as the process progressed, she integrated more closely into the activities. Detailed observations of verbal and non-verbal interactions were made and recorded in a field journal immediately after each observation session. Outside the study area, these observations were described descriptively, avoiding subjective interpretations, and maintaining a balance between immersion and objectivity.

### Data Analysis

Data analysis was conducted simultaneously with data collection, following the principles of ethnography. Observations made of the participants allowed for the identification of patterns, which were recorded in an Excel template and subsequently compared with the analysis derived from the interviews.

Once conducted, the interviews were transcribed by two research assistants and provided to the participants to share the preliminary findings and improve the quality and validity of the research. Independently, the three researchers conducted initial coding after reading the transcriptions multiple times. Subsequently, the codes were compared, merged, and discussed to reach a consensus, resolving any disagreements after several rounds of analysis. As codes were grouped, three themes and six sub-themes emerged, following the framework proposed by Braun and Clarke for thematic analysis ^27^. Once the results were developed, they were presented to the participants for review, and they were accepted without suggestions.

### Ethical Aspects

The study received approval from the ethics committee of Universidad Surcolombiana according to minutes 002 of May 17, 2022. Data confidentiality was ensured through the anonymization of the transcriptions and the assignment of a code to identify the participants.

## RESULTS

Twenty caregivers of children with multiple disabilities participated, and Table 1 presents their characterization. The following themes emerged from the analysis: a) Social support network: integration, information sharing, and mutual assistance; b) Learning space: learning to care for oneself and others; c) Empowerment promotion: identifying and addressing access barriers (Table 2).

**Table 1:** Sociodemographic characterization of caregivers of children with multiple disabilities.

		N=20	%
Age			
	25-29	9	45.0
	30-34	5	25.0
	35-39	2	10.0
	40-44	2	10.0
	Over 45 years old	2	10.0
Educational level			
	Primary school	6	30.0
	Basic Secondary	8	40.0
	High School Education	4	20.0
	Technologist	2	10.0
Marital status			
	Single	3	15.0
	Married	4	20.0
	Common-law marriage	3	15.0
	Separated	10	50.0
Socioeconomic level			
	Low	19	95.0
	Medium	1	5.0
Occupation			
	Unemployed	16	80.0
	Self-employed	4	20.0

**Table 2:** Themes and testimonies from interviews and focus groups

Themes	Testimonial
Social support network: integrating, informing, and mutually helping each other	*“That’s how the group was born, in that going back and forth alone, from here to there, the need arose to come together and form a group to share information and help each other” (Mireya)* *“When I joined, the first thing I thought was: this is where I should be, these moms have children like my daughter, here they won’t look at my daughter like a freak” (Paola)* *“(...) we had to come together, together we are stronger (...)” (Virginia)* *“We are organized into groups according to the type of disability our children have, we are the caregivers of children with multiple disabilities” (Mireya)* *“The group has helped us a lot to get informed (...) we have learned to understand how services work, how to claim treatments” (Maruja)* *“I try not to miss the meetings, because here they inform us about government programs and if you don’t participate, you don’t find out anything” (Pepa)* *“We are not alone, we connect and keep each other informed all the time through WhatsApp, if I need any kind of help, if I can find a medication, if they opened appointments, if they know a certain doctor (...), if they can accompany or help me with the child” (Camila)* *“I don’t have my family... dad, mom, siblings here in Neiva..., we left everything (...), I only have, let’s say, this group, here I have had a lot of support for everything related to my daughter” (Andrea)*
Learning space: learning to care for oneself and others	*“Here I have understood more about my daughter’s situation, her growth is slower, and I no longer compare her to other children, now I see that she eats well, holds her head, sits, and crawls on the floor and that motivates me, that has served me a lot” (Virginia)* *“I feel like I’m where I belong here, other children may be in the same or worse situation than my son, they all have their process and I have learned to handle it” (Inés)* *“My daughter was not eating, I had to give her a gastrostomy (…), but I found another mom in the same situation and you learn from that other person, how she does it, what she prepares for her and my daughter has improved a lot, she doesn’t get sick anymore” (Pepa)* *“I have met mothers of children who also have seizures, I have learned more (…), for example, how to prevent choking on secretions, I feel more confident now” (Cielo)* *“Now I understand more my role as a caregiver, the group has helped me control myself more, that anger and frustration I held (…), also the psychologist who supports us, but here you get distracted and learn both the good and the bad that one has and how to improve it” (Inés)* *“I look forward to the day of the meeting to forget about everything, I unload completely, then I come home calmer and stronger” (Luna)*
Promotes empowerment: identifying and confronting barriers to access	*“I have benefited a lot from the talks with the psychologists and the workshops that have been held (…), I keep demanding, but I don’t lose control, I insist and with information in hand, I assert my rights” (Inés)* *“Having a child with a disability has also been an opportunity for us women to get to know ourselves and see what we are capable of” (Angela)* *“We have had to take control, inform ourselves about the regulations” (Lina)* *“Now I know that there is an ombudsman, the personería, or the ombudsman’s office where we can claim the rights that children have” (Perla)* *“I started with the ombudsman (…), they told me well, that we had to form a watchdog group, we got involved in the watchdog group (…), we learned a little more about the health sector, we did a very interesting job with the ombudsman (…)” (Mireya)* *“We have also been in the disability committee, in short, to fight for our rights we have participated in everything” (Inés)* *“As ‘Discapacidad Huila’ we are networked with Redescol and we participated in the signing of the pact for inclusion” (Mireya)*

### a) Social support network: integrating, informing, and assisting each other.

The support group spontaneously emerged from successive encounters with six individuals who were individually experiencing the same crises, situations, and uncertainties related to caring for their children. They shared the same fears, stress, and suffering, so they decided to come together to share information and support each other.

They identified with each other because they shared the same discrimination and stigmatization due to their children’s condition, which to the eyes of “others” were not “normal”. They felt that their lives had changed, so as mothers with “different” children, they decided to encourage other people in similar situations to join or form other groups that now function as a network identified as “Disability Huila”. They firmly believe that unity is strength and that together they can be more visible to the society that discriminates against them.

Participating in the support group has allowed them to stay informed, understand, and clarify medical information and administrative procedures. This information has helped reduce worry, fear, anxiety, and distress about their children’s condition and the complexity of the healthcare system. In the support group, they receive firsthand information about social and economic support programs for their families because the group is recognized at the municipal level.

The women feel they are no longer alone; they have found others who are experiencing similar situations. Their peers are an important source of support for the multiple needs they face. They communicate continuously through a WhatsApp group where they can freely express their fears and anxieties, seek guidance on practical aspects of caregiving, and offer and receive help as needed. For some caregivers, the group is their only source of support due to forced displacement from rural to urban areas to access healthcare services for their children.

### b) Learning space: learning to care for oneself and others.

The support group has helped them understand and accept their children’s health condition and assume their care with greater tranquility. They have learned to act in different caregiving situations, to face the challenges of treatment, and to focus more on valuing their children’s achievements and abilities rather than their disabilities.

They mention that they were not prepared to take on the parenting of their children; collectively, they have been able to learn and/or improve their caregiving skills, resolve doubts regarding technical information provided by healthcare personnel, initially difficult to understand, but clarified by their peers based on experience. With the training provided by nurses, psychologists, and social workers, they have managed to resolve doubts and learned techniques and procedures they were unfamiliar with. Now they feel greater security and confidence and consider that they have achieved better caregiving outcomes and well-being for their children.

They have learned to take care of themselves. They are more aware of their role as caregivers, their strengths and weaknesses, and how to enhance their self-care abilities. In the support group meetings, they can rest, reduce their stress, and alleviate the burden of caregiving. For some women, this is the only leisure or recreational space they have.

### c) Promotes empowerment: identifying and addressing access barriers.

The support group has facilitated a process of reflection and self-awareness through workshops led by support professionals, where strengths and weaknesses have been identified at both individual and group levels, and the leadership and empowerment of caregivers have been promoted. In this process, they decided to educate themselves about the regulations that protect their rights and the available tools to take control and confront the access barriers they encounter.

Based on the tools learned, they have supported each other and made requests, complaints, and/or claims to health, education, and governmental institutions responsible for monitoring and controlling services for their children. The application of these tools has benefited them individually and collectively.

Thanks to their participation and development of leadership skills in the group, they have had the opportunity to serve as monitors of health programs, engage in social control spaces, and support the Municipal Health Department in the preparation of the Registry for the Localization and Characterization of Persons with Disabilities (RLCPD).

The support group is part of the departmental network of “Disability Huila” and the National Network of networks of people with disabilities for Colombia - Redescol, which in June 2022 signed a National Pact for the Inclusion of people with disabilities, their families, and family caregivers, thus initiating a process of social pressure to fight for the well-being and quality of life of caregivers. In January 2022, they participated in the first meeting of caregivers CICI (International Collective of Comprehensive Caregivers).

## DISCUSSION

Qualitatively, the perceived benefits of a support group consisting of 20 women caregivers of children with multiple disabilities are explored. Its members have a strong sense of belonging, strong bonds of friendship, diversity of roles, and function integrated into a network that they originated and call “Disability Huila”, recognized for its commitment to initiatives in favor of the population with disabilities.

They perceive themselves as a social support network since they are the main source of information they have to reduce the uncertainty generated by the complexity of the healthcare system where they attend to their children. Other studies have pointed out how support groups offer valuable information to parents of children with rare diseases and decrease their uncertainty after navigating through complicated healthcare systems ^28^. Additionally, they provide practical information about caregiving and how to navigate and confront organizational challenges, becoming a complementary support to healthcare services ^29^.

The support group facilitates coping and resilience following the birth and upbringing of a child with multiple disabilities, a similar outcome to that reported in a meta-analysis where a positive and significant association was found between resilience and social support in family caregivers of children with autism ^30^. During critical moments, responsibilities associated with the care of their children and themselves are shared, with solidarity and mutual assistance coexisting, as seen in caregivers of children with complex needs ^31^. Platforms such as WhatsApp allow them to stay informed, and request and offer assistance, as documented in support groups for caregivers of children with neurodisabilities ^32^.

This space allows for collective learning, encouraging a different approach to parenting that values children’s achievements over their disabilities. Here, mothers can resolve practical concerns and gain greater confidence in caring for their children. Evidence shows that support groups led by experienced mothers of children with Congenital Zika Syndrome disabilities provide a beneficial and well-received approach. These groups promote sharing, learning, mutual support, and encouragement ^33^. They fill information gaps that health services may leave for caregivers of people with Parkinson’s disease ^34^, facilitate the exchange of personal experiences among mothers of autistic children ^35^, and equip mothers of children with multiple disabilities due to congenital Zika syndrome to manage common health problems ^19^.

The support group promotes caregivers’ interest in self-care, encourages learning about self-care, and improves their physical and psychological well-being, as evidenced in support groups for caregivers of children with developmental disabilities ^36^.

Similarly, they contribute to disconnecting and reducing the caregiving burden, as noted in support groups for children with intellectual disabilities ^37^. The high degree of participation and social cohesion achieved in the group has also contributed to the mental health of its members, supporting findings from studies that have found that the well-being of caregivers of children with developmental disabilities tends to be higher when there are high levels of integration or social support ^22^.

Clearly, they promote the empowerment of caregivers to identify and address access barriers through a process of reflection, awareness, self-discovery, and learning guided by support professionals; this has allowed them to take control of imposed barriers and set precedents that benefit the group as a whole. These benefits have also been associated with the implementation of community-based inclusive development strategies for caregivers of children with learning and developmental disabilities, confirming that there is an association between participation in self-help groups and increased personal agency or empowerment in caregivers of children with disabilities ^38^.

Despite the study’s limitations, such as the inability to generalize results due to convenience sampling and the inability to transfer results to other settings as it was conducted in a city in southern Colombia, it can be concluded that support groups for women caregivers of children with multiple disabilities are a valuable tool that provides them with emotional support, practical information, and coping strategies, promotes their empowerment, and helps them develop caregiving and self-care skills. Additionally, these groups contribute to improving the quality of life and well-being of both the children and the caregivers. It is relevant to highlight the importance of these groups as a source of social support and as an effective way to promote caregiver empowerment, inclusion, and advocacy for the rights of children with disabilities.

Furthermore, the active participation of healthcare professionals and other support professionals in coordinating and facilitating the support group is highlighted, contributing to its sustainability and quality over time. Sustained commitment and a shared vision are required for the effective implementation of these support groups, thus helping to prevent the violation of the fundamental rights of children with disabilities. Therefore, it is a valuable intervention that should be integrated into the Community-Based Rehabilitation (CBR) Strategy for the social inclusion of people with disabilities.

## References

[1] Olusanya BO, Boo NY, Nair MKC, Samms-Vaughan ME, Hadders-Algra M, Wright SM (2022). Accelerating progress on early childhood development for children under 5 years with disabilities by 2030. Lancet Glob Health.

[2] N'Dri KM, Yaya I, Zigoli R, Endemel Ayabakan F, Ipou SY, Lambert Moke B (2018). Impact du polyhandicap de l'enfant sur les familles à Abidjan. Sante Publique (Paris).

[3] World health Organization Disability 2023.

[4] Pan American Health Organization (2019). 57th Directing Council 71st Session of the regional committee of who for the americas.

[5] Kuper H, Heydt P (2019). The Missing Billion.

[6] Cubillos Alzate JC, Perea Caro SA (2020). Boletines poblacionales: Personas con Discapacidad Diciembre 2020.

[7] United Nations Children's Fund (2021). Seen, Counted, Included.

[8] Bright T, Wallace S, Kuper H (2018). A Systematic Review of Access to Rehabilitation for People with Disabilities in Low- and Middle-Income Countries. Int J Environ Res Public Health.

[9] Ministerio de Salud y Protección Social (2022). Lineamientos generales para la implementación de la Política Pública Nacional de Discapacidad e Inclusión Social en entidades territoriales 2013 - 2022.

[10] Batista Conceição dos Santos D, Vázquez-Ramos V, da Costa Cunha Oliveira C, López-Arellano O (2019). Accesibilidad en salud revisión sobre niños y niñas con discapacidad en Brasil-Perú-Colombia. Rev Latinoam Cienc Soc Niñez Juv.

[11] Zuurmond M, Nyante G, Baltussen M, Seeley J, Abanga J, Shakespeare T (2019). A support programme for caregivers of children with disabilities in Ghana Understanding the impact on the wellbeing of caregivers. Child Care Health Dev.

[12] Farhadi A, Bahreini M, Moradi A, Mirzaei K, Nemati R (2022). The predictive role of coping styles and sense of coherence in the post-traumatic growth of mothers with disabled children a cross-sectional study. BMC Psychiatry.

[13] Freitas PSS, Soares GB, Mocelin HJS, Lamonato LCXL, Sales CMM, Linde-Arias AR (2020). How do mothers feel Life with children with congenital Zika syndrome. Int J Gynecol Obstet.

[14] Marquis SM, McGrail K, Hayes M (2020). Mental health of parents of children with a developmental disability in British Columbia, Canada. J Epidemiol Community Health.

[15] Asa GA, Fauk NK, Ward PR, Mwanri L (2020). The psychosocial and economic impacts on female caregivers and families caring for children with a disability in Belu District, Indonesia. PLoS One.

[16] Bourke-Taylor HM, Joyce KS, Grzegorczyn S, Tirlea L (2022). Profile of Mothers of Children with a Disability Who Seek Support for Mental Health and Wellbeing. J Autism Dev Disord.

[17] Lovell B, Elder GJ, Wetherell MA (2021). Sleep disturbances and physical health problems in caregivers of children with ASD. Res Dev Disabil.

[18] Yoosefi lebni J, Ziapour A, Khosravi B, Rahimi khalifeh kandi Z (2021). Lived experience of mothers of children with disabilities a qualitative study of Iran. J Public Health (Berl).

[19] Laza-Vásquez C, Gea-Sánchez M, Briones-Vozmediano E (2022). Qualitative evaluation of a support group for women with children with Zika congenital syndrome in Southern Colombia. Disabil Rehabil.

[20] Williams NA, Villachan-Lyra P, Hatton-Bowers H, Marvin C, Chaves E, Hollist C (2023). Family-Centered Practices and Caregiver Mental Health in a Developmental Intervention for Young Children With Congenital Zika Syndrome. Infants Young Child.

[21] Ministerio de Salud y Protección Social (2014). Lineamientos Nacionales De Rehabilitación Basada En La Comunidad - RBC.

[22] Dembo RS, Huntington N, Mitra M, Rudolph AE, Lachman ME, Mailick MR (2022). Social network typology and health among parents of children with developmental disabilities Results from a national study of midlife adults. Soc Sci Med.

[23] Rashid M, Caine V, Goez H The Encounters and Challenges of Ethnography as a Methodology in Health Research.

[24] Hammersley M, Atkinson P (2019). Ethnography: Principles in Practice.

[25] Etikan I (2016). Comparison of Convenience Sampling and Purposive Sampling. American Journal of Theoretical and Applied Statistics.

[26] Tenny S, Brannan JM, Brannan GD (2024). Qualitative Study. StatPearls.

[27] Braun V, Clarke V (2019). Reflecting on reflexive thematic analysis. Qual Res Sport Exerc Health.

[28] Kenny T, Bogart K, Freedman A, Garthwaite C, Henley S, Bolz-Johnson M (2022). The importance of psychological support for parents and caregivers of children with a rare disease at diagnosis. Rare Dis Orphan Drugs J.

[29] Joo JH, Bone L, Forte J, Kirley E, Lynch T, Aboumatar H (2022). The benefits and challenges of established peer support programmes for patients, informal caregivers, and healthcare providers. Fam Pract.

[30] Iacob CI, Avram E, Cojocaru D, Podina IR (2020). Resilience in Familial Caregivers of Children with Developmental Disabilities A Meta-analysis. J Autism Dev Disord.

[31] Klein O, Walker C, Aumann K, Anjos K, Terry J (2022). Peer support groups for parent-carers of children with attention deficit hyperactivity disorder the importance of solidarity as care. Disabil Soc.

[32] Mohamed IN, Elseed MA (2021). Utility of WhatsApp in healthcare provision and sharing of medical information with caregivers of children with neurodisabilties experience from Sudan. Sudan J Paediatr.

[33] Smythe T, Matos M, Reis J, Duttine A, Ferrite S, Kuper H (2020). Mothers as facilitators for a parent group intervention for children with Congenital Zika Syndrome Qualitative findings from a feasibility study in Brazil. PLoS One.

[34] Fothergill-Misbah N, Moffatt S, Mwithiga H, Hampshire K, Walker R (2022). The role of support groups in the management of Parkinson's disease in Kenya Sociality, information and legitimacy. Glob Public Health.

[35] Lamba N, Van Tonder A, Shrivastava A, Raghavan A (2022). Exploring challenges and support structures of mothers with children with Autism Spectrum Disorder in the United Arab Emirates. Res Dev Disabil.

[36] Chafouleas SM, Iovino EA, Koriakin TA (2020). Caregivers of Children with Developmental Disabilities Exploring Perceptions of Health-Promoting Self-Care. J Dev Phys Disabil.

[37] Leyva-López A, Rivera-Rivera L, Márquez-Caraveo ME, Toledano-Toledano F, Saldaña-Medina C, Chavarría-Guzmán K (2022). Estudio de la calidad de vida en cuidadores familiares de personas con discapacidad intelectual. Salud Publica Mex.

[38] Bunning K, Gona JK, Newton CR, Andrews F, Blazey C, Ruddock H (2020). Empowering self-help groups for caregivers of children with disabilities in Kilifi, Kenya Impacts and their underlying mechanisms. PLoS One.

