# Supplementary material for: Benefits of being part of a support group for caregivers of children with multiple disabilities: a qualitative study
Source: Rev Peru Med Exp Salud Publica. 2024 Mar 27;41(1):54–61. doi: 10.17843/rpmesp.2024.411.13218 (PMC11149755; doi:10.17843/rpmesp.2024.411.13218)
Supplement: Translation

# Translation: Beneficios de pertenecer a un grupo de apoyo de cuidadoras de niños con discapacidad múltiple: un estudio cualitativo

**Authors:** María Elena Rodríguez-Vélez, Claudia Patricia Cantillo-Medina, Alix Yaneth Perdomo-Romero

**Affiliations:** 1 Universidad Surcolombiana. Facultad de Salud, Programa de Enfermería. Huila, Colombia.

**Keywords:** Grupo de Autoayuda, Niños con Discapacidad, Apoyo Social, Enfermería Comunitaria, Investigación cualitativa

## Abstract

**Objetivo.:**

Explorar la percepción de los beneficios de participar en un grupo de apoyo de cuidadoras de niños con discapacidad múltiple.

**Materiales y métodos.:**

Estudio cualitativo, enfoque etnográfico focalizado, realizado de octubre del 2022 a febrero del 2023, muestreo por conveniencia, participaron 20 cuidadoras de niños con discapacidad múltiple. La información se recolectó mediante observación participante, grupos de discusión y entrevistas semiestructuradas. Se realizó análisis temático aplicando las propuestas por Braun y Clark.

**Resultados.:**

Los temas emergentes fueron: red de apoyo social: integrarse, informarse y ayudarse mutuamente; espacio de aprendizaje: se aprende a cuidar y a cuidarse; promueve el empoderamiento: identificar y enfrentar barreras de acceso.

**Conclusiones.:**

El grupo de apoyo funciona como una red de apoyo social, proporciona información, reduce la incertidumbre, facilita el afrontamiento y la resiliencia tras el nacimiento y crianza de un hijo con discapacidad múltiple. Espacio donde se aprende a cuidar y a cuidarse a sí mismo y donde se promueve el empoderamiento para la defensa de los derechos de los niños con discapacidad.

## INTRODUCCIÓN

La probabilidad de que un niño tenga una discapacidad antes de cumplir cinco años es diez veces mayor que la probabilidad de morir ^1^. Los niños con discapacidad múltiple presentan múltiples expresiones de discapacidad, siempre asociadas a deterioro motor e intelectual que los colocan en una posición de alta dependencia ^2^. La discapacidad infantil representa un desafío significativo a nivel global ^3^, siendo reconocida como un problema de salud pública y social, con repercusiones en la calidad de vida de millones de niños y sus cuidadoras ^4^. En todo el mundo, se estima que un número considerable de niños enfrentan algún tipo de discapacidad, lo que afecta su capacidad para participar plenamente en la sociedad y acceder a oportunidades educativas, de salud y sociales adecuadas ^5^.

En América Latina, incluyendo Colombia, la situación de la discapacidad infantil presenta desafíos específicos. Si bien, hay avances en términos de legislación y políticas de inclusión, muchas familias enfrentan dificultades para acceder a servicios de salud y educación adecuados para sus hijos con discapacidad. Además, la discriminación y el estigma social siguen siendo barreras significativas que afectan la vida diaria de estos niños y sus cuidadores ^3^. En Colombia, el Huila figura entre los departamentos con mayor proporción de personas con discapacidad y los niños representan un 8% de esta población ^6^.

Los niños en condición de discapacidad están en desventaja en la mayoría de las medidas de bienestar infantil, tienen acceso limitado a los servicios de salud y rehabilitación en países de bajos y medianos ingresos ^7^ y hay escasez de profesionales de la salud capacitados para atenderlos ^8^. En efecto, en nuestro país a pesar de contar con una Política Pública de Discapacidad e Inclusión Social ^9^ persisten dificultades de acceso a servicios básicos de educación, salud y protección social para estos niños, agravadas en años recientes debido a la pandemia de COVID-19 ^10^.

La familia tiene una importancia crucial para garantizar el desarrollo de los niños en condición de discapacidad ^11^ pero enfrenta diversas presiones psicológicas, sociales y económicas ^12^ que aumentan si el niño tiene una discapacidad múltiple ^13^. El impacto psicológico es mayor en las mujeres, que deben enfrentar separaciones o divorcios, asumir exclusivamente el rol de cuidadoras y padecer estigma social ^14^; se sienten inferiores, inseguras y estresadas por las dificultades económicas ^15^; presentan síntomas depresivos en rango clínico ^16^ y trastornos del sueño ^17^; son frecuentes los sentimientos de culpa, pérdida de oportunidades, sacrificio materno, preocupación por el futuro y falta de apoyo socioeconómico ^18^.

Los grupos de apoyo (GA) son una alternativa de red de apoyo social; fomentan la comprensión de la situación, las competencias de cuidado, reducen el estigma social y el aislamiento ^11^ son fuente de información, apoyo emocional y relacional para mujeres con niños con discapacidad ^19^, favorecen la resiliencia, el afrontamiento familiar, la salud y bienestar de los cuidadores ^20^. Recientemente son considerados en nuestro país como parte del componente de empoderamiento de la Estrategia de Rehabilitación Basada en Comunidad (RBC) para la inclusión social de las personas con discapacidad ^21^. A pesar de estos beneficios, la evidencia señala que los padres de niños con discapacidad tienen restricción en redes de apoyo o no cuentan con ellas, en comparación con los padres de niños sin discapacidad, por lo que diferentes investigaciones instan a los profesionales de la salud hacia la necesidad de apoyar o implementar este tipo de estrategias ^22^.

Adicionalmente, se evidencia la escasez de investigaciones centradas específicamente sobre los beneficios percibidos de los GA para cuidadores de niños con discapacidad múltiple en América Latina, incluyendo Colombia. Aunque existen estudios que exploran el impacto de los GA en cuidadores de niños con discapacidad en general, hay una falta de comprensión detallada sobre cómo estos grupos benefician específicamente a aquellos que cuidan a niños con discapacidades múltiples en entornos de países en desarrollo como Colombia. Teniendo en cuenta lo anterior y la conformación de un GA de cuidadores de niños con discapacidad múltiple en el sur de Colombia que ha permanecido vigente durante seis años y ha evolucionado hasta ser reconocido a nivel municipal e incluso departamental, se realizó un estudio cualitativo etnográfico para explorar la percepción de los beneficios de participar en un grupo de apoyo de cuidadoras de niños con discapacidad múltiple, con el fin de comprender cómo estas dinámicas influyen en su experiencia de cuidado y bienestar.

**MENSAJE CLAVE**

Motivación para realizar el estudio: Llenar un vacío de conocimiento sobre los grupos de apoyo de cuidadoras de niños con discapacidad múltiple.

Principales hallazgos: Los grupos de apoyo son espacios valiosos de apoyo social y aprendizaje para las cuidadoras de niños con discapacidad múltiple. Facilitan la resiliencia, el afrontamiento tras tener un niño con discapacidad múltiple. Favorecen la participación y empoderamiento de las cuidadoras de niños con discapacidad múltiple para enfrentar barreras de acceso y defender los derechos fundamentales de los niños.

Implicancias: Es importante fomentar y respaldar los grupos de apoyo para cuidadoras de niños con discapacidad múltiple.

## MATERIALES Y MÉTODOS

### Diseño de estudio

Estudio de etnografía focalizada, diseño contemporáneo enfocado en los pequeños elementos de la sociedad y en las vidas de las personas que están “social y culturalmente fragmentadas y diferenciadas”; centrándose en el estudio de las experiencias compartidas de un fenómeno más limitado y predeterminado de pequeños grupos sociales para comprender problemas sociales específicos, la interrelación entre las personas y el contexto social en que viven y comparten los participantes, sus perspectivas sobre los eventos y cuestiones sociales ^23^.

La información se recopiló desde octubre de 2022 a febrero de 2023 al sur de Colombia, con un GA de cuidadoras de niños con Discapacidad Múltiple que hacen parte de una red de apoyo denominada “Discapacidad Huila”. El primer paso en la investigación fue establecer contacto con la líder de la red, quien actúo como portera de campo ^24^.

### Equipo de investigación

El equipo de investigación estuvo compuesto por tres investigadoras que tenían experiencia en estudios con diseños cualitativos. Las investigadoras estaban vinculadas como docentes de la Universidad Surcolombiana al momento de realizar el estudio.

### Contexto

El GA “Discapacidad Huila”, ha sido una iniciativa vital en la región durante los últimos seis años. Surgió como respuesta a la creciente necesidad de apoyo y orientación para las cuidadoras que enfrentan los desafíos únicos asociados con la crianza de niños con discapacidades múltiples en el contexto local. Fundado en 2018 por un pequeño número de cuidadoras que buscaban compartir experiencias, recursos y brindarse apoyo mutuo. El GA, ha experimentado un crecimiento significativo tanto en tamaño como en reconocimiento, expandiéndose de un grupo informal a una red de apoyo bien establecida y reconocida a nivel municipal e incluso departamental.

Actualmente, el GA está compuesto aproximadamente por 30 cuidadoras comprometidas y activas que desempeñan un papel vital en la comunidad de cuidadores de niños con discapacidad en la región del Huila y ha tenido un impacto significativo en la comunidad local, al mejorar la calidad de vida de sus miembros, así como concientizar al ente gubernamental sobre las necesidades y desafíos enfrentados por las familias de niños con discapacidad en la región.

### Selección de los participantes

Se realizó un muestreo por conveniencia ^25^, participaron 20 cuidadoras de niños menores de 9 años con discapacidad múltiple, que cumplieron con los criterios de ser mayores de 18 años, mínimo cuatro años de pertenecer al GA y aceptar voluntariamente participar en el estudio. No hubo personas que se negaran a participar del estudio.

### Recolección de información

Se utilizó una ficha para caracterizar los participantes. Las investigadoras interactuaron en el entorno de las participantes con el fin de observar sus actividades en su contexto natural. Así mismo, se invitaron a un aula de la universidad para conformar dos grupos de discusión con 10 cuidadoras cada uno y duración aproximada de 90 minutos, con el fin de realizar una inmersión y exploración inicial de los puntos de vista colectivos sobre el tema y definir, junto con la revisión de la literatura, los temas para las entrevistas semiestructuradas. Posteriormente en el lugar de reunión del GA se llevaron a cabo, veinte entrevistas semiestructuradas y la observación participante ^26^, actividades realizadas por las investigadoras con experiencia en las técnicas de recolección en la metodología definida. No se realizaron entrevistas repetidas.

Previo a la obtención de la información se explicó a los participantes la metodología y se solicitó la firma del consentimiento para participar en el estudio y grabar el audio de las entrevistas. La siguiente fue la pregunta norteadora “¿Qué opinan ustedes sobre la funcionalidad del GA, su experiencia en el grupo y beneficios percibidos?”. A partir de esta pregunta, se generó la discusión y la construcción del discurso, por el grupo, sobre el tema “beneficios de pertenecer a un grupo de apoyo”.

Asimismo, y después del análisis de la información obtenida en cada grupo de discusión, se entrevistó individualmente a los participantes en una sola ocasión, para ampliar y profundizar en ciertos temas de interés emergentes. Esto, como una alternativa de triangulación de datos que permitió conseguir una mayor riqueza, amplitud y profundidad de información, y credibilidad de los resultados. Las entrevistas se realizaron hasta no encontrar nuevos hallazgos, con lo cual se consideró la saturación para el análisis planteado. Los grupos de discusión y las entrevistas fueron grabados en audio y transcritas íntegramente en las siguientes 48 horas. Las entrevistas transcritas se devolvieron a los participantes para su lectura y aprobación con lo descrito.

La observación participante ^26^^)^ fue llevada a cabo de manera gradual por la investigadora principal, inicialmente observando a cierta distancia, a medida que avanzaba el proceso, la investigadora se fue integrando un poco más a las actividades, se realizaron observaciones detalladas de las interacciones verbales y no verbales, las cuales se registraban en un diario de campo, inmediatamente después de cada sesión de observación; fuera del terreno de estudio se redactaba de manera descriptiva, evitando interpretaciones subjetivas, manteniendo un equilibrio entre inmersión y objetividad.

### Análisis de los datos

El análisis de datos se llevó a cabo de manera simultánea con la recolección de los mismos, siguiendo los principios de la etnografía. Las observaciones realizadas a los participantes permitieron la identificación de patrones, los cuales fueron registrados en una plantilla en Excel y posteriormente comparados con el análisis derivado de las entrevistas.

Las entrevistas, una vez realizadas, fueron transcritas por dos auxiliares de investigación y entregadas a los participantes con el fin de compartir los hallazgos preliminares, enriquecer y mejorar la calidad y validez de la investigación. Las tres investigadoras de forma independiente luego de leer en varias ocasiones las transcripciones realizaron una primera codificación. Posteriormente, los códigos se compararon, fusionaron y discutieron para llegar a un consenso, y en casos de desacuerdos, se resolvieron después de varias rondas de análisis. A medida que los códigos se agruparon surgieron tres temas y seis subtemas, siguiendo el marco propuesto por Braun y Clarke para el análisis temático ^27^. Una vez elaborados los resultados, se presentaron a las participantes para su revisión y estos fueron aceptados sin sugerencias.

### Aspectos éticos

El estudio recibió aval del comité de ética de la Universidad Surcolombiana según acta 002 del 17 de mayo del 2022, se garantizó la confidencialidad de los datos mediante la anonimización de las transcripciones y asignación de un número para identificar a las participantes.

## RESULTADOS

Participaron 20 cuidadoras de niños con discapacidad múltiple, la Tabla 1 presenta su caracterización. Al realizar el análisis emergen los siguientes temas, a) Red de apoyo social: integrarse, informarse y ayudarse mutuamente; b) Espacio de aprendizaje: se aprende a cuidar y a cuidarse; c) Promueve el empoderamiento: identificar y enfrentar barreras de acceso (Tabla 2).

**Tabla 1:** Caracterización sociodemográfica de las cuidadoras de niños con discapacidad múltiple.

		N=20	%
Edad			
	25-29	9	45,0
	30-34	5	25,0
	35-39	2	10,0
	40-44	2	10,0
	Mayor de 45 años	2	10,0
Nivel de escolaridad			
	Primaria	6	30,0
	Básica Secundaria	8	40,0
	Educación Media	4	20,0
	Tecnólogo	2	10,0
Estado civil			
	Soltera	3	15,0
	Casada	4	20,0
	Unión libre	3	15,0
	Separada	10	50,0
Nivel socioeconómico			
	Bajo	19	95,0
	Medio	1	5,0
Ocupación			
	Desempleada	16	80,0
	Independiente	4	20,0

**Tabla 2:** Temas y testimonios de las entrevistas y grupos de discusión

Temas	Testimonio
Red de apoyo social: integrarse, informarse y ayudarse mutuamente	*“así nació el grupo, en ese ir y venir solas, de aquí para allá, nació la necesidad de unirnos y de conformar un grupo para compartirnos la información y para ayudarnos unas con otras”* (Mireya) *“Cuando ingresé, lo primero que pensé fue: aquí es donde debo estar, estas mamás tienen niños como mi hija, aquí no van a mirar a mi hija como un fenómeno”* (Paola) “*(…) había que unirse, unidas somos más (…)”* (Virginia) *“estamos organizados por grupos según el tipo de discapacidad de nuestros hijos, nosotras somos las cuidadoras de niños con discapacidad múltiple”* (Mireya) *“El grupo nos ha ayudado mucho para informarnos (…) hemos aprendido a conocer cómo funcionan los servicios, como reclamar los tratamientos”* (Maruja) *“Yo trato de no faltar a las reuniones, porque aquí nos informan de los programas del gobierno y si uno no participa pues no se entera de nada”* (Pepa) *“Nosotras no estamos solas, nos conectamos y mantenemos informadas todo el tiempo por WhatsApp, que si necesito cualquier tipo de ayuda, que donde consigo un medicamento, si abrieron agenda, si conocen tal médico (…), que si me pueden acompañar o ayudar con el niño”* (Camila) *“Yo no tengo mi familia… papá, mamá, hermanos aquí en Neiva., dejamos todo (…), solo tengo, digámoslo así, a este grupo, aquí he tenido mucho apoyo para todo lo de mi niña”* (Andrea)
Espacio de aprendizaje: se aprende a cuidar y a cuidarse	*“Acá he comprendido más la situación de mi hija, el crecimiento de ella es más lento, ya no la comparo con otros niños, ahora veo que ella me come bien, me sostiene la cabeza, se sienta, se arrastra por el piso y eso me motiva, eso me ha servido mucho”* (Virginia) *“Yo siento que aquí estoy donde es, otros niños pueden estar igual o peor que mi hijo, todos tienen su propio proceso y he aprendido a manejarlo”* (Inés) *“Mi hija ya no mandaba, le toco la gastrostomía, (…), pero encontré otra mamá con la misma situación y uno aprende de esa otra persona, como lo hace, que le prepara y es mucho lo que mi hija ha mejorado, ya no se me enferma”* (Pepa) *“He conocido a madres de niños que también convulsionan, he aprendido más (…), por ejemplo, cómo hacer para que no se me ahogue con las secreciones, ya me siento más segura”* (Cielo) *“Ahora comprendo más mi papel de cuidadora, el grupo me ha servido para controlarme más, esa rabia y frustración que mantenía (…), también la psicóloga que nos apoya, pero pues uno acá se distrae y aprende también lo bueno y lo malo que una tiene y como puede mejorarlo”* (Inés) *“Yo espero el día de la reunión para olvidarme de todo, me descargo totalmente, después llego a mi casa más tranquila y con más fuerzas”* (Luna)
Promueve el empoderamiento: identificar y enfrentar barreras de acceso	*“Me han servido mucho las charlas con las psicólogas y los talleres que se han hecho (…), sigo reclamando, pero no pierdo el control, insisto y con información en la mano hago valer mis derechos”* (Inés) *“Tener un hijo con discapacidad también ha sido una oportunidad para conocernos y ver de lo que somos capaces nosotras las mujeres”* (Angela) *“Nos ha tocado como el cuento tomar el control, informarnos sobre las normas”* (Lina) “*Ahora sé que existe una contraloría, la personería o la defensoría del pueblo donde podemos reclamar los derechos que tienen los niños”* (Perla) *“Yo empecé con la contraloría (…), me dijeron bueno que hay que hacer un grupo de veeduría, nos metimos a la veeduría (…), aprendimos un poco más del sector salud, hicimos un trabajo bien interesante con la contraloría (…)”* (Mireya) *“También hemos estado en el comité de discapacidad, en fin, por luchar por nuestros derechos hemos participado en todo”* (Inés) *“Como Discapacidad Huila estamos en red con Redescol y participamos en la firma del pacto por la inclusión”* (Mireya)

### Red de apoyo social: integrarse, informarse y ayudarse mutuamente.

El grupo de apoyo nació de manera espontánea tras encuentros sucesivos de seis personas que pasaban en solitario las mismas crisis, situaciones e incertidumbre relacionadas con la atención de sus hijos, compartían los mismos temores, estrés y sufrimiento, por lo que decidieron unirse para compartir información y ayudarse mutuamente.

Se identificaban entre sí, porque compartían la misma discriminación y estigmatización por la condición de sus hijos, que a la vista de los “otros” no eran “normales”. Sentían que sus vidas habían cambiado, entonces como madres con niños “diferentes” decidieron motivar a otras personas en su misma situación para que se unieran o formaran otros grupos que en la actualidad funcionan como una red identificada como “Discapacidad Huila”. Creen firmemente que la unión hace la fuerza y que juntas pueden ser más visibles ante la sociedad que las discrimina.

Participar en el GA les ha permitido mantenerse informadas, comprender y clarificar información médica y trámites administrativos. Esta información ha contribuido a disminuir la preocupación, temor, ansiedad y angustia por la condición de sus hijos y la complejidad del sistema de salud. En el GA reciben de primera mano información sobre programas de apoyo social y económico para sus familias porque el grupo es reconocido a nivel municipal.

Las mujeres sienten que ya no están solas, han encontrado a otras personas que viven experiencias similares, sus pares son una fuente importante de apoyo ante las múltiples necesidades que presentan. Se comunican permanentemente a través de un grupo de WhatsApp donde pueden expresar libremente sus miedos y angustias, solicitar orientación sobre aspectos prácticos del cuidado, pedir y brindar ayuda según necesidad. Para algunas cuidadoras, el grupo es su única fuente de apoyo debido al desplazamiento forzoso del campo a la ciudad por la alta demanda de servicios de salud de sus hijos.

### b) Espacio de aprendizaje: se aprende a cuidar y a cuidarse.

El GA les ha ayudado a comprender y aceptar la condición de salud de sus hijos y asumir su cuidado con mayor tranquilidad, han aprendido a actuar frente a las diferentes situaciones de cuidado que se les presentan, a enfrentar los retos del tratamiento y a preocuparse más por valorar los logros y capacidades, más que la discapacidad de sus hijos.

Mencionan que no estaban preparadas para asumir la crianza de sus hijos; en colectivo han podido aprender y/o mejorar sus habilidades de cuidado, resolver dudas respecto a información técnica suministrada por personal de salud, inicialmente complicada de entender, pero clarificada por sus pares con base en la experiencia. Con las capacitaciones brindadas por enfermeras, psicólogos y trabajadoras sociales han logrado resolver dudas y aprendido técnicas y procedimientos que desconocían. Ahora sienten mayor seguridad y confianza y consideran que han logrado mejores resultados de cuidado y bienestar de sus hijos.

Han aprendido a cuidar de sí mismas. Son más conscientes de su rol de cuidadoras, sus fortalezas y debilidades y como potenciar sus habilidades de autocuidado. En las reuniones del GA pueden descansar, disminuir su estrés y aliviar la carga del cuidado. Para algunas mujeres, este es el único espacio de ocio o esparcimiento.

### c) Promueve el empoderamiento: identificar y enfrentar barreras de acceso.

El GA ha facilitado un proceso de reflexión y autoconocimiento a través de talleres liderados por profesionales de apoyo, donde se han identificado fortalezas y debilidades a nivel individual y grupal y promovido el liderazgo y empoderamiento de las cuidadoras. En este proceso decidieron informarse sobre las normas que amparan sus derechos y herramientas disponibles para tomar el control y enfrentar las barreras de acceso que se les presentan.

Con base en las herramientas aprendidas se han apoyado mutuamente y realizado peticiones, quejas y/o reclamos ante instituciones de salud, educación y gubernamentales encargadas de la vigilancia y control de servicios para sus hijos. La aplicación de estas herramientas les ha beneficiado individual y colectivamente.

Gracias a su participación y desarrollo de habilidades de liderazgo en el grupo, han tenido la oportunidad de participar como veedoras de programas de salud, actuar en espacios de control social y apoyar a la Secretaría de Salud Municipal con la elaboración del Registro para la Localización y Caracterización de las Personas con Discapacidad (RLCPD).

El GA hace parte de la red departamental de “Discapacidad Huila” y de la Red Nacional de redes de personas con discapacidad para Colombia-Redescol, que en junio del 2022 firmó un Pacto Nacional por la Inclusión de personas con discapacidad, sus familias y cuidadores familiares, iniciando de esta manera un proceso de presión social para luchar por el bienestar y calidad de vida de las cuidadoras. En enero del 2022 participaron en la realización del primer encuentro de cuidadoras CICI (Colectivo Internacional de Cuidadoras Integrales).

## DISCUSIÓN

Se exploran cualitativamente los beneficios percibidos de GA conformado por 20 mujeres cuidadoras de niños con discapacidad múltiple. Sus integrantes tienen alto sentido de pertenencia, fuertes lazos de amistad, diversidad de roles y funcionan integradas a una red a la que dieron origen y denominan “Discapacidad Huila”, reconocida por su compromiso con iniciativas en favor de la población en condición de discapacidad.

Se perciben como red de apoyo social ya que son la principal fuente de información que poseen para reducir la incertidumbre generada por la complejidad del sistema de salud donde atienden a sus hijos. Otros estudios han señalado como los GA ofrecen información valiosa a padres de niños con enfermedades raras y disminuyen su incertidumbre tras su recorrido por sistemas de salud complicados ^28^, además brindan información práctica sobre el cuidado y como navegar y enfrentar los desafíos organizacionales, convirtiéndose en un apoyo complementario de los servicios de salud ^29^.

El GA facilita el afrontamiento y la resiliencia tras el nacimiento y crianza de un hijo con discapacidad múltiple; resultado similar al reportado en un metaanálisis en donde se encontró una asociación positiva y significativa entre la resiliencia y el apoyo social en cuidadores familiares de niños con autismo ^30^. En momentos críticos, se comparten responsabilidades asociadas al cuidado de sus hijos y de sí mismos, coexistiendo la solidaridad y mutua ayuda, como sucede en cuidadores de niños con necesidades complejas ^31^ Plataformas como WhatsApp les permite mantenerse informadas, solicitar y ofrecer ayuda, como se ha registrado en GA de cuidadores de niños con neurodiscapacidad ^32^.

Es un espacio de aprendizaje colectivo que ayuda a asumir la crianza de manera diferente, valorando logros más que discapacidades; donde pueden resolver dudas prácticas y ganar mayor seguridad y confianza respecto al cuidado que brindan a sus hijos. Se ha evidenciado que el apoyo de madres cuidadoras con experiencia en el cuidado de niños con discapacidad asociado al Síndrome de Zika Congénito es un enfoque beneficioso y aceptable que promueve compartir, aprender, apoyarse y animarse unos a otros ^(^^33^, llena vacíos de información que no suministran los servicios de salud en cuidadores de personas con Parkinson ^34^, posibilita el intercambio de información basada en experiencias personales en madres de niños con autismo ^35^ y enseña a manejar problemas de salud comunes entre madres de niños con discapacidad múltiple por síndrome de Zika congénito ^19^.

El GA promueve el interés de las cuidadoras en el cuidado de sí mismas, fomenta el aprendizaje sobre el autocuidado y mejora su bienestar físico y psicológico, tal como se ha evidenciado en GA de cuidadoras de niños con discapacidad de desarrollo ^(^^36^.

Así mismo, contribuyen a desconectarse y disminuir la carga de cuidado como ha sido señalado en GA de niños con discapacidad intelectual ^37^. El alto grado de participación y cohesión social logrado en el grupo ha contribuido igualmente a la salud mental de sus integrantes, respaldando resultados de estudios que han encontrado que el bienestar de los cuidadores de niños con discapacidad del desarrollo tiende a ser mayor cuando existen altos grados de integración o apoyo social ^22^.

Evidentemente, promueven el empoderamiento de las cuidadoras para identificar y enfrentar barreras de acceso, a través de un proceso de reflexión, concientización, autoconocimiento y aprendizaje orientado por profesionales de apoyo; esto les ha permitido tomar el control de las barreras impuestas y crear precedentes que benefician al grupo en general. Estos beneficios también han sido asociados con la implementación de estrategias de desarrollo inclusivo basado en comunidad de cuidadores de niños con discapacidad del aprendizaje y del desarrollo, confirmando que existe asociación entre la participación en grupos de autoayuda y una mayor agencia personal o empoderamiento en cuidadores de niños con discapacidades ^(^^38^.

A pesar de las limitaciones del estudio consistentes en no poder generalizar los resultados debido al empleo de un muestreo por conveniencia y no poder transferir los resultados a otros entornos por haberse realizado en una ciudad al sur de Colombia, se puede concluir que los GA para mujeres cuidadoras de niños con discapacidad múltiple son una herramienta valiosa que les proporciona apoyo emocional, información práctica y estrategias de afrontamiento, fomenta su empoderamiento y les ayuda a desarrollar habilidades de cuidado y autocuidado. Además, estos grupos contribuyen a mejorar la calidad de vida y el bienestar de los niños y de las cuidadoras. Es relevante destacar la importancia de estos grupos como fuente de apoyo social y como forma efectiva de promover el empoderamiento de las cuidadoras, la inclusión y defensa de los derechos de los niños con discapacidad.

Además, se destaca la participación activa de los profesionales de la salud y otros profesionales de apoyo en la coordinación y facilitación del GA, aportando a su sostenibilidad y calidad en el tiempo. Se requiere de un compromiso sostenido y una visión compartida para la implementación efectiva de estos GA, contribuyendo de esta manera a prevenir la vulneración de los derechos fundamentales de los niños con discapacidad, por lo que es una valiosa intervención que debe articularse a la Estrategia de Rehabilitación Basada en Comunidad (RBC) para la inclusión social de las personas con discapacidad.
